# Who are we reaching? Identifying subgroups among individuals seeking help for opioid use disorder

**DOI:** 10.3389/fpsyt.2026.1753193

**Published:** 2026-03-09

**Authors:** Carla Faßbender, Carlotta Riemerschmid, Monika Murawski, Ursula Berger, Eva Hoch, Larissa Schwarzkopf

**Affiliations:** 1 IFT Institut für Therapieforschung, Centre for Mental Health and Addiction Research, Munich, Germany; 2 Institute for Medical Information Processing, Biometry, and Epidemiology (IBE), Ludwig-Maximilians-University, Munich, Germany; 3 Pettenkofer School of Public Health, Ludwig-Maximilians-University, Munich, Germany; 4 Department of Psychiatry and Psychotherapy, LMU University Hospital, LMU Munich, Munich, Germany; 5 Bavarian Association of Statutory Health Insurance Physicians, Munich, Germany; 6 Division of Clinical Psychology and Psychotherapy, Charlotte-Fresenius-University, Munich, Germany

**Keywords:** comorbid substance use disorder, injection drug use, latent class analysis, opioid use disorder, outpatient addiction care facilities

## Abstract

**Introduction:**

Opioid use disorder (OUD) represents a major challenge in addiction care, yet empirical insights into the sociodemographic, clinical, and care-related profiles of help-seekers remain limited. Understanding these profiles is essential for developing targeted care strategies. This study therefore aims to identify and describe latent classes of help-seekers with OUD and outline their specific needs.

**Methods:**

Latent Class Analysis was applied to routinely collected person-level data from the 2023 Berlin Addiction Care Statistical Service to identify latent classes of outpatient help-seekers with OUD. Comorbid substance use disorders (SUDs) and injection drug use served as indicators. Classes were compared across sociodemographic, clinical, and care-related variables using Bonferroni-adjusted χ²-tests.

**Results:**

Among 2,833 help-seekers, three latent classes were identified: *Individuals Primarily Using Opioids* (n = 1,381), *Individuals with Multiple SUDs* (n = 709), and *Individuals Who Inject Drugs* (n = 743). *Individuals Primarily Using Opioids* did not exhibit clearly distinctive patterns of service utilization and disproportionately reported methadone or other opioids as their main substances of use. *Individuals with Multiple SUDs* were characterized by high social stability (e.g., partnerships, independent living) and a high enrolment in opioid agonist treatment (OAT). *Individuals Who Inject Drugs* were marked by heightened vulnerability, including precarious housing and elevated rates of HIV and hepatitis C and primarily accessed low-threshold services.

**Discussion and conclusion:**

The results highlight the heterogeneity of help-seekers with OUD and emphasize the need for targeted, class-specific care strategies. *Individuals Primarily Using Opioids* warrant further investigation, as this class may still comprise heterogeneous subgroups with diverse care needs. *Individuals with Multiple SUDs* may benefit from more flexible OAT frameworks that accommodate work and family responsibilities. For *Individuals Who Inject Drugs*, integrated health and social services, combined with expanded harm reduction efforts (e.g., syringe exchange, testing opportunities for infectious diseases), may help reduce access barriers and effectively address their complex needs.

## 1 Introduction

Opioids, including natural, synthetic, and semi-synthetic substances, have a high addictive potential and are associated with severe individual and societal harms (1, 2). Globally, an estimated 16.2 million individuals were affected by opioid use disorder (OUD) in 2021, resulting in 11.2 million disability-adjusted life years (DALYs) and nearly 100,000 deaths, underscoring persistent increases in prevalence and disease burden over recent decades (3, 4).

Over the past three decades, the global public health burden of OUD has increased markedly—particularly among young adults aged 15–24 and in countries with a high sociodemographic index (5). This trend is particularly pronounced in the United States, where the “opioid crisis” has led to steep increases in overdose deaths and a growing treatment demand (6). While there is currently no evidence suggesting that Europe is on the verge of a similar crisis (7, 8), research points to an increasing prevalence of prescription opioid (mis)use in several European countries (9, 10) and a shift toward more frequent use of synthetic opioids (11).

Despite increasing prevalence rates and changing patterns of use, pan-European treatment monitoring systems continuously report stable or even declining rates of opioid-related treatment admissions in several countries, including Germany (12). This discrepancy suggests that current treatment offers do not reach all individuals with OUD—potentially due to barriers to care or unmet needs. Individuals with OUD frequently report stigma, concerns about privacy, negative expectations regarding treatment effectiveness, or emotional readiness as obstacles to seeking help (13, 14). In addition, structural barriers such as limited service availability, long waiting times, insufficiently trained providers, and legal restrictions on pharmacological treatment further constrain access to care (15).

To address these challenges and the diverse needs of individuals with OUD, Germany offers a highly differentiated and largely cost-free addiction care system, including low-threshold services (e.g., consumption rooms, emergency shelters), counseling centers, outpatient clinics, opioid agonist treatment (OAT), and long-term rehabilitation (16). However, it remains unclear which subgroups are effectively addressed by this system. While international evidence has highlighted the heterogeneity of individuals with OUD and substance use patterns more broadly (17–22), little is known about how this heterogeneity manifests among help-seeking individuals within the addiction care system.

By focusing on help-seeking individuals with OUD, this study advances understanding of which profiles are currently reached by addiction services, offering evidence to better tailor interventions to clients’ needs.

Against this background, this study aims to:

1. Identify latent classes within the help-seeking population based on diagnoses of comorbid substance use disorders (SUDs) at admission and injection drug use within the last 12 months.
2. Outline and compare the sociodemographic characteristics, care-related factors, consumption patterns, and health profiles of these classes.

## 2 Methods

### 2.1 Data source

Data stem from the 2023 Berlin Addiction Care Statistical Service (in German: *Berliner Suchthilfestatistik [BSHS]*), a part of Germany’s addiction-related treatment demand monitoring system. Data of *N* = 55 participating outpatient addiction care facilities (OACFs), representing 82% of all OACFs in Berlin, were analyzed.

Within BSHS, collection of sociodemographic, clinical, and care-related characteristics of the help-seeking population follows the German Core Dataset (in German: *Deutscher Kerndatensatz [KDS]*), a nationwide documentation standard aligned with the pan-European protocol for the Treatment Demand Indicator (23, 24). Documentation is case-based, meaning that each newly initiated care episode—whether in the same or a different facility—is counted as a separate entry (“case”). A person-specific identifier allows the assignment of multiple cases to a unique help-seeker.

Data collection adheres to the Declaration of Helsinki (revised in 2013) as well as regional (Berlin Data Protection Law), national (German Data Protection Law), and international (European General Data Protection Regulation) data protection requirements. Informed consent is obtained from all help-seekers, whose data are included in BSHS.

### 2.2 Setting and study sample

The initial sample comprised 29,841 case-based admissions. To focus on individuals with current OUD, the sample was limited to cases fulfilling at least one of the following criteria:

▪  A diagnosis of OUD reflects either harmful use (F11.1) or dependence (F11.2) according to the International Statistical Classification of Diseases and Related Health Problems, 10th Revision (ICD-10, German modification) (25). With reference to the date of admission, the diagnosis of “harmful use” requires that the pattern of use had persisted for at least four weeks or had recurred within the past 12 months. For a diagnosis of dependence, the reference period was four weeks prior to assessment (25). (26).
▪ A diagnosis of polysubstance use disorder [ICD-10 codes: F19.1 or F19.2; (25)] with reported opioid use within the 30 days prior to admission. This reflects the growing body of evidence that individuals with OUD frequently engage in simultaneous or sequential use of multiple psychoactive substances (27–29).
▪ Consumption of opioids (heroin, methadone, buprenorphine, fentanyl, other opioids) as the primary substance at the time of admission.
▪ Participation in opioid agonist treatment (OAT) or receipt of psychosocial care during OAT.

The sample was further restricted to individuals with a valid person-specific identifier, aged between 10 and 99 years, and at least two contacts with the respective OACF, one of which occurred in 2023. This requirement ensures that sufficient data are available for analysis, as detailed information in outpatient care is typically collected from the second contact onward. To avoid double counting of individuals with multiple cases, only the most recent case was retained. A detailed description of the sample selection process is provided in the supplement (see **Supplementary Figure 1**).

### 2.3 Latent class analysis

To identify subgroups among help-seekers with OUD, a Latent Class Analysis (LCA) was conducted in accordance with an overview of methodological guidance and best practice recommendations (30, 31). LCA is a probabilistic, model-based approach designed to identify latent (unobserved) classes based on individuals’ shared response patterns to categorical indicator variables (31). This method is particularly valuable for uncovering latent classes that could benefit from tailored care approaches informed by shared characteristics (32).

### 2.4 Indicator variables for LCA

Prior research on consumption patterns has highlighted the high prevalence of injection drug use (33) and the concurrent use of substances such as cocaine, cannabinoids, alcohol, and tobacco among individuals with OUD (27–29). Building upon this evidence, we focused on diagnoses of comorbid SUDs to ensure co-substance use at a clinically relevant level and incorporated injection drug use as an additional indicator for enhanced clinical complexity, as done in prior latent class analyses summarized in a recent systematic review (34). The following indicator variables were selected:

▪ Diagnosis of comorbid alcohol use disorder (F10.1, F10.2), cannabinoid use disorder (F12.1, F12.2), cocaine use disorder (F14.1, F14.2), tobacco use disorder (F17.1, F17.2), or polysubstance use disorder (F19.1, F19.2) at the time of admission in dummy-coded format (yes/no) (25).
▪ Self-reported injection drug use within the last 12 months, as a categorical variable (yes/no/missing).

To reduce the likelihood of identifying a class based on a single variable, only indicators with a prevalence ≥10% in the sample were included (30). Accordingly, the diagnosis of comorbid stimulant use disorder was excluded due to its low prevalence (8.2%).

Associations between indicator variables were examined using Cramér’s V to ensure local independence, both for the full sample and within each class. Variables with Cramér’s V exceeding the recommended threshold of 0.5 were excluded (30).

The main analysis included only cases without missing values in the indicator variables, which deviates from the general recommendation to perform LCAs using a Full Information Maximum Likelihood (FIML) approach. This was a deliberate decision, as the pattern of missing data was evidently missing not at random, violating the missing-at-random assumption underlying FIML (30). To test the robustness of the results, two sensitivity analyses were conducted. In the first analysis (SA1), missing values for injection drug use were treated as a separate category to assess their potential impact on model outcomes. In the second analysis (SA2), comorbid stimulant use disorder (F15.1, F15.2) was added as an additional indicator variable, given its growing prevalence and associated risks among individuals with OUD (12).

### 2.5 Evaluation of the LCA models

To determine the optimal number of classes, a series of latent class models ranging from one to six classes was estimated and evaluated based on goodness-of-fit indices and classification quality.

#### 2.5.1 Goodness-of-fit indices

The Bayesian Information Criterion (BIC) and the Sample-adjusted BIC (SABIC) were used to assess model fit, with lower values indicating a better balance between model accuracy and parsimony (30, 31). These indices were visualized using an elbow plot illustrating the improvements in fit by adding further classes (see **Supplementary Figure 2**).

#### 2.5.2 Model characteristics and classification accuracy

Model evaluation further considered the smallest class size, entropy, and average posterior probabilities. Determining the smallest class size at 10% ensures that each class contains enough individuals for meaningful interpretation and adequate statistical power. Entropy values (ranging from 0 to 1) were used to assess class separation, with higher values indicating clearer distinctions between classes. Classification certainty was evaluated using average posterior probabilities, summarized in a classification matrix. Diagonal values reflect the average probability of correctly assigning individuals to their most likely class, with values closer to 1 indicating more accurate classification. Values above 0.70 indicate acceptable classification, values above 0.80 suggest good posterior classification, and values above 0.90 indicate excellent classification certainty (30, 31).

#### 2.5.3 Final model selection and class assignment

The final latent class solution was selected based on goodness-of-fit indices, classification accuracy measures, and substantive interpretability to ensure a robust and meaningful classification. Each individual was assigned to the class with the highest posterior probability, applying a threshold of ≥ 0.5 to ensure accurate assignment (30). Cases below this threshold were excluded from subsequent analyses.

### 2.6 Variables selected for class comparison

In an additional step, sociodemographic, care-related, consumption, and health variables were analyzed to characterize the identified classes and assess differences between them. To maintain clarity and focus on the research question, only the most relevant categories of each variable are presented.

Sociodemographic variables addressed biological sex (“female”), age at admission (“≤29 years”; “30–50 years”; “>50 years”), education level (“school dropout”), employment status (“unemployed”), living situation (“precarious housing” (including homelessness or temporary shelters); “independent living”), first-generation migration background (“yes”), partnership (“yes”) and parenthood (“yes”).

Care-related variables addressed first-time admission to addiction care (“yes”), referral pathway to OACF (“medical and addiction treatment facilities”), OAT (“yes”) and primary treatment (“low-threshold facilities”; “psychosocial intervention alongside OAT”; “addiction and drug counseling”). The OAT variable was defined as binary, assuming “yes” when explicitly coded and “no” in all other cases.

Consumption and health variables addressed shared syringe use (“within the last 12 months”), HIV, hepatitis B (HBV), and hepatitis C (HCV) testing (“within the last 12 months”), test results (“positive”), the primary substance of use (“proportion of opioids overall”; “heroin”; “methadone”; “buprenorphine”; “fentanyl”; “other opioids”), and self-reported heroin use in the 30 days prior to admission (mean number of days of use).

Missing values were reported for all variables.

To examine differences between classes, we conducted hierarchical testing using χ²-tests. For each categorical variable, a global χ²-test across all classes was performed to assess overall significance. Variables showing significant global results were then analyzed in *post-hoc* pairwise χ²-tests to identify which class pairs differed. To account for multiple comparisons across the 24 categorical variables, a Bonferroni correction was applied, yielding a corrected significance level of 0.002 (α = 0.05/24).

All analyses were conducted using the statistical software RStudio (Version 2024.09.1 + 394; Posit Software, PBC, formerly RStudio, PBC; 250 Northern Ave, Suite 420, Boston, MA 02210) (35). The LCA was implemented using the *poLCA* package (36).

## 3 Results

### 3.1 Sample characteristics and prevalence of indicator variables

The analysis sample comprised 3,021 individuals with current OUD. In the main analysis, 158 individuals were excluded due to missing values in the indicator variable for injection drug use, resulting in a final sample of 2,863.

The most prevalent indicator variable was self-reported injection drug use (33.8%; see **Table 1**), followed by diagnoses of comorbid cocaine use disorder (28.6%) and polysubstance use disorder (23.1%). Diagnoses of comorbid cannabinoid use disorder (20.6%), tobacco use disorder (19.1%), and alcohol use disorder (18.4%) were less prevalent.

**Table 1:** Prevalence of indicator variables.

Indicator variables	n	%
Injection Drug Use within the last 12 months	968	33.8
Comorbid Cocaine Use Disorder	819	28.6
Comorbid Polysubstance Use Disorder	660	23.1
Comorbid Cannabinoid Use Disorder	584	20.4
Comorbid Tobacco Use Disorder	546	19.1
Comorbid Alcohol Use Disorder	526	18.4

N = 2,863.

The indicator variables showed only weak to moderate associations with one another (Cramér’s V < 0.5), indicating low multicollinearity and supporting their suitability as indicator variables for identifying latent classes.

### 3.2 Evaluation of models and final model selection

The four-class model showed the lowest BIC and SABIC but only marginally outperformed the three-class model (see **Table 2**). In the three-class solution, the smallest class comprised 25% of the sample, ensuring sufficient class sizes. Additionally, this model demonstrated higher entropy than the four-class model while also being more parsimonious. The average posterior probabilities ranged from 0.79 (Class 1; “acceptable”) over 0.85 (Class 3: “good”) to 0.96 (Class 2; “excellent”). Based on these considerations, the three-class model was selected.

**Table 2:** Goodness-of-fit indices and average posterior probabilities of investigated models.

Number of classes	Goodness-of-fit indices		Average posterior probabilities for each class						Entropy	Smallest class	
No.	BIC	SABIC	C1	C2	C3	C4	C5	C6	-	n	%
1	18481.1	18476.9	1	–	–	–	–	–	–	2,863	100
2	17685.6	17676.6	0.91	0.91	–	–	–	–	0.62	996	35.8
3	**17627.9**	**17614.0**	**0.79**	**0.96**	**0.85**	–	–	–	**0.63**	**716**	**25.0**
4	17597.4	17578.7	0.73	0.68	0.79	0.88	–	–	0.55	310	10.8
5	17622.4	17598.8	0.85	0.66	0.76	0.79	0.85	–	0.63	201	7.0
6	17648.4	17620.0	0.79	0.85	0.60	0.74	0.92	0.78	0.61	125	4.4

N = 2,863. BIC, Bayesian Information Criterion; SABIC, Sample-Adjusted BIC; C, Class. Bold font indicates selected model.

The five- and six-class models were not considered further, as they included classes comprising fewer than 10% of the sample. Sensitivity analyses (SA1 and SA2) confirmed the robustness of the results by showing similar class solutions (see **Supplementary Figures 3**, **4**).

### 3.3 Class description

**Figure 1:**
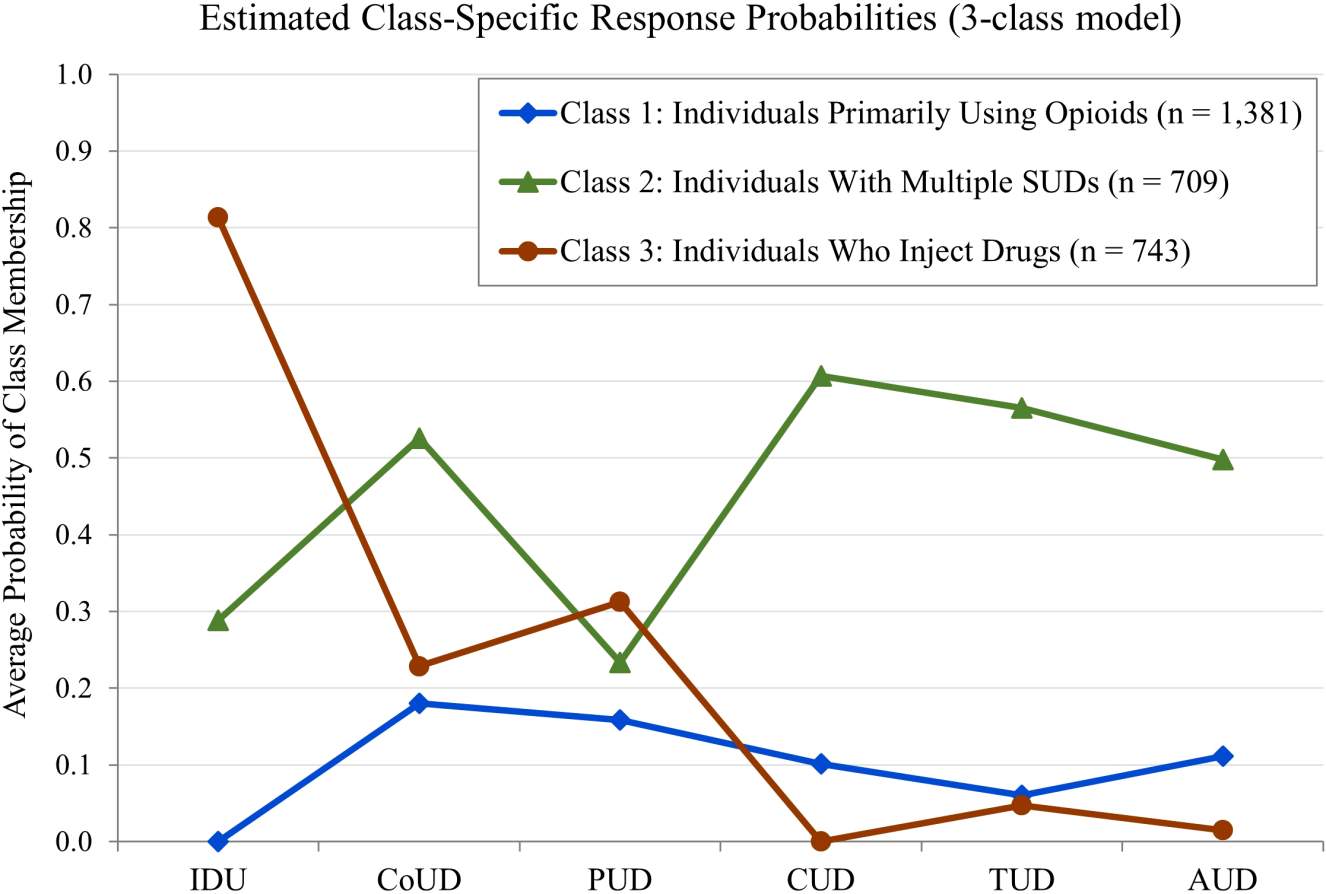
Estimated class-specific response probabilities for indicator variables, based on the three-class model. Sample Size was N = 2,833. IDU, Injection Drug Use; CoUD, Cocaine Use Disorder; PUD, Polysubstance Use Disorder; CUD, Cannabinoid Use Disorder; TUD, Tobacco Use Disorder; AUD, Alcohol Use Disorder. Higher scores represent a high probability of a particular indicator variable.

**Figure 1** illustrates the estimated class-specific response probabilities for the indicator variables within the three-class model. Details on the two- and four-class models are provided in the supplement (see **Supplementary Figures 5**, **6**).

Thirty cases with posterior probabilities below the 0.5 threshold could not be reliably assigned to any class, resulting in a final sample of N = 2,833 for further analysis. Classes were labeled based on their most distinctive indicator variables.

Class 1 (48.8%, n = 1,381) was characterized by non-injection use and low probabilities of comorbid SUDs and was labeled *Individuals Primarily Using Opioids*. Class 2 (25.0%, n = 709) exhibited a moderate probability of injection drug use and comparatively high probabilities of distinct comorbid SUDs and was labeled *Individuals with Multiple SUDs*. Class 3 (26.2%, n = 743) showed by far the highest probability of injection drug use, combined with low to moderate probabilities of comorbid SUDs, and was labeled *Individuals Who Inject Drugs.*

### 3.4 Characteristics of classes and their comparisons

Significant differences were identified across all sociodemographic variables; therefore, pairwise comparisons between classes were conducted (see **Table 3**). *Individuals with Multiple SUDs* were more likely to be women and aged ≤29 years, whereas *Individuals Primarily Using Opioids* were more likely to be aged ≥50 years. Unemployment and school dropout were most common among *Individuals with Multiple SUDs.* This group also had a lower proportion of individuals with a first-generation migration background and a higher proportion living in a partnership compared with the other two classes. *Individuals Who Inject Drugs* were less likely to be parents or to live independently but were more likely to experience precarious housing compared to the other classes.

**Table 3:** Sociodemographic characteristics of extracted classes.

	Total sample (N = 2,833)		Class 1 *individuals primarily using opioids* (48.8%, n = 1,381)		Class 2 *individuals with multiple SUDs* (25.0%, n = 709)		Class 3 *individuals who inject drugs* (26.2%, n = 743)		Global p-value	Pairwise comparisons by hierarchical testing
Variables and Response Categories	n	%	n	%	n	%	n	%	–	–
Biological sex										
Female	**553**	19.5	**276**	20.0	**161**	22.7	**116**	15.6	p < 0.002	C2 ≠ C3
NA	**10**	0.4	**2**	0.1	**5**	0.7	**3**	0.4		
Age at admission										
Up to 29 years	**673**	23.8	**332**	24.0	**201**	28.3	**140**	18.8	p < 0.002	C2 ≠ C3
30-50 years	**1.721**	60.7	**816**	59.1	**396**	55.9	**509**	68.5		
Above 50 years	**439**	15.5	**233**	16.9	**112**	15.8	**94**	12.7	p < 0.002	C1 ≠ C3
NA	**0**	0	**0**	0	**0**	0	**0**	0		
Level of education										
School dropout	**340**	12.0	**173**	12.5	**126**	17.8	**41**	5.5	p < 0.002	C1 ≠ C2 ≠ C3
Other	**1.548**	54.6	**773**	56.0	**568**	80.1	**207**	27.9		
NA	**945**	33.4	**435**	31.5	**15**	2.1	**495**	66.6		
Employment status										
Unemployed	**907**	32.0	**431**	31.2	**347**	48.9	**129**	17.4	p < 0.002	C1 ≠ C2 ≠ C3
Other	**1.019**	36.0	**549**	39.8	**349**	49.2	**121**	16,3		
NA	**907**	32.0	**401**	29.0	**13**	1.8	**493**	66.4		
Living situation										
Precarious housing	**488**	17.2	**203**	14.7	**90**	12.7	**195**	26.2	p < 0.002	C3 ≠ C1, C2
Independent living	**1,108**	39.1	**553**	40.0	**325**	45.8	**230**	31.0	p < 0.002	C3 ≠ C1, C2
Other	**1,158**	40.9	**579**	41.9	**292**	41.2	**287**	38.6		
NA	**79**	2.8	**46**	3.3	**2**	0.3	**31**	4.2		
First-generation migration background										
Yes	**1,095**	38.7	**568**	41.1	**178**	25.1	**349**	47.0	p < 0.002	C2 ≠ C1, C3
NA	**197**	7.0	**95**	6.9	**21**	3.0	**81**	10.9		
Partnership										
Yes	**578**	20.4	**307**	22.2	**205**	28.9	**66**	8.9	p < 0.002	C1 ≠ C2 ≠ C3
NA	**918**	32.4	**406**	29.4	**18**	2.5	**494**	66.5		
Parenthood										
Yes	**598**	21.1	**312**	22.6	**205**	28.9	**81**	10.9	p < 0.002	C3 ≠ C1, C2
NA	**928**	32.8	**410**	29.7	**23**	3.2	**495**	66.6		

N = 2,833. NA = Missing Value.

“Other” includes additional categories of the variable that are not reported in detail here.

To account for multiple comparisons across the 24 categorical variables, a Bonferroni correction was applied, yielding a corrected significance level of 0.002 (α = 0.05 / 24), indicated by "≠" within the table.

For example, for the variable 'living situation', Class 3 significantly differs from both Class 1 and Class 2 in the proportion of individuals with precarious housing situation. This is noted as C3 ≠ C1, C2. Bold font used for easier readability.

Regarding care-related variables (see **Table 4**), *Individuals with Multiple SUDs* had the lowest likelihood of first-time admissions. *Individuals Who Inject Drugs* were less likely to be referred by medical or addiction treatment facilities and less likely to participate in OAT than the other two classes. *Individuals with Multiple SUDs* were more likely to receive psychosocial interventions alongside OAT than the others. *Individuals Who Inject Drugs* were most likely to utilize low-threshold services and less likely to access addiction and drug counseling.

**Table 4:** Care-related factors of extracted classes.

	Total Sample (N = 2,833)		Class 1 *individuals primarily using opioids* (48.8%, n = 1,381)		Class 2 *individuals with multiple SUDs* (25.0%, n = 709)		Class 3 *individuals who inject drugs* (26.2%, n = 743)		Global p-value	Pairwise comparisons by hierarchical testing
Variables and Response Categories	**n**	%	**n**	%	**n**	%	**n**	%	–	–
First-time admission										
Yes	**755**	26.7	**393**	28.5	**142**	20.0	**220**	29.6	p < 0.002	C2 ≠ C1, C3
*NA*	***147***	*5.2*	***90***	*6.5*	***9***	*1.3*	***48***	*6.5*		
Referral pathway to OACFs										
Medical/Addiction Treatment Facility	**392**	13.8	**198**	14.3	**124**	17.5	**70**	9.4	p < 0.002	C3 ≠ C1, C2
Other	**1,467**	51.8	**749**	54.2	**549**	77.4	**169**	22.7		
*NA*	***974***	*34.4*	***434***	*31.4*	***36***	*5.1*	***504***	*67.8*		
Opioid agonist treatment										
Yes	**577**	20.4	**296**	21.4	**177**	25.0	**104**	14.0	p < 0.002	C3 ≠ C1, C2
Primary treatment intervention										
Low-threshold facilities	**923**	32.6	**388**	28.1	**46**	6.5	**489**	65.8	p < 0.002	C1 ≠ C2 ≠ C3
Psychosocial Intervention alongside OAT	**370**	13.1	**159**	11.5	**141**	19.9	**70**	9.4	p < 0.002	C2 ≠ C1, C3
Addiction and drug counseling	**1,460**	49.6	**785**	56.8	**442**	62.3	**179**	24.1	p < 0.002	C3 ≠ C1, C2

N = 2,833. NA, Missing Value; OACFs, Outpatient Addiction Care Facilities; OAT, Opioid Agonist Treatment.

“Other” includes additional categories of the variable that are not reported in detail here.

To account for multiple comparisons across the 24 categorical variables, a Bonferroni correction was applied, yielding a corrected significance level of 0.002 (α = 0.05 / 24), indicated by "≠" within the table.

For example, for the variable 'referral pathway to OACFs', Class 3 significantly differs from both Class 1 and Class 2 in the proportion of referral through medical/addiction treatment facilities. This is noted as C3 ≠ C1, C2. Bold font used for easier readability.

In terms of consumption patterns and health profiles (see **Table 5**), *Individuals Who Inject Drugs* had a higher likelihood of shared syringe use and of undergoing HIV, HBV, and HCV testing within the past 12 months. Additionally, the prevalence of positive HIV or HCV test results was significantly higher compared to *Individuals Primarily Using Opioids*.

**Table 5:** Consumption patterns and health profiles of extracted classes.

	Total sample (N = 2,833)		Class 1 *individuals primarily using opioids* (48.8%, n = 1,381)		Class 2 *individuals with multiple SUDs* (25.0%, n = 709)		Class 3 individuals who inject drugs (26.2%, n = 743)		Global p-value	Pairwise comparisons by hierarchical testing
Variables and Response Categories	**n**	%	**n**	%	**n**	%	**n**	%	–	–
Shared syringe use										
Within last 12 months	**99**	3.5	**7**	0.5	**28**	3.9	**64**	8.6	p < 0.002	C1 ≠ C2 ≠ C3
*NA*	***1,512***	*53.4*	***1,009***	*73.1*	***421***	*59.4*	***82***	*11.0*		
HIV test										
Within last 12 months	**986**	41.4	**387**	34.7	**232**	38.5	**367**	55.2	p < 0.002	C3 ≠ C1, C2
*NA*	***449***	*15.8*	***265***	*19.2*	***106***	*15.0*	***78***	*10.5*		
HIV test results										
Positive	**90**	3.2	**25**	1.8	**19**	2.7	**46**	6.2	p < 0.002	C1 ≠ C3
*NA*	***1,210***	*42.7*	***670***	*48.5*	***322***	*45.4*	***218***	*29.3*		
HBV test										
Within last 12 months	**959**	33.9	**379**	27.4	**221**	31.2	**359**	48.3	p < 0.002	C3 ≠ C1, C2
*NA*	***480***	*16.9*	***278***	*20.1*	***110***	*15.5*	***92***	*12.4*		
HBV test results										
Positive	**63**	2.2	**27**	2.0	**16**	2.3	**20**	2.7	Non-sig.	
*NA*	***1,545***	*54.6*	***842***	*61.0*	***390***	*55.0*	***314***	*42.3*		
HCV test										
Within last 12 months	**983**	34.7	**386**	28.0	**229**	32.3	**368**	49.5	p < 0.002	C3 ≠ C1, C2
*NA*	***465***	*16.4*	***274***	*19.8*	***108***	*15.2*	***83***	*11.2*		
HCV test results										
Positive	**231**	8.2	**69**	5.0	**70**	9.9	**92**	12.4	p < 0.002	C1 ≠ C2; C3
*NA*	***1,460***	*51.5*	***820***	*59.4*	***373***	*52.6*	***267***	*35.9*		
Primary substance										
Proportion of opioids overall	**1,870**	66.0	**971**	70.3	**403**	56.8	**496**	66.8	p < 0.002	C2 ≠ C1; C3
Heroin	**1,115**	39.4	**518**	37.5	**230**	32.4	**367**	49.4		
Methadone	**292**	10.3	**183**	13.3	**69**	9.7	**40**	5.4		
Buprenorphine	**99**	3.5	**47**	3.4	**30**	4.2	**22**	3.0		
Fentanyl	**13**	0.5	**8**	0.6	**2**	0.3	**3**	0.4		
Other Opioids	**351**	12.4	**215**	15.6	**72**	10.2	**64**	8.6		
*NA*	***21***	*0.7*	***12***	*0.9*	***1***	*0.1*	***8***	*1.1*		
Heroin use in the last 30 days										
*Mean days of use*	***14.1***		***14.0***		***9.1***		***19.7***			
*NA*	***845***	*29.8*	***469***	*34.0*	***241***	*34.0*	***135***	*18.2*		

N = 2,833. NA, Missing Value; Non-sig, Non-significant; HIV, Human Immunodeficiency Virus; HBV, Hepatitis B; HCV, Hepatitis C.

To account for multiple comparisons across the 24 categorical variables, a Bonferroni correction was applied, yielding a corrected significance level of 0.002 (α = 0.05 / 24), indicated by "≠" within the table.

For example, for the variable 'HIV Test', Class 3 significantly differs from both Class 1 and Class 2 in the proportion of individuals who received an HIV test within the last 12 months. This is noted as C3 ≠ C1, C2. Bold font used for easier readability.

## 4 Discussion

This study assigned help-seekers with OUD in three distinct classes: *Individuals Primarily Using Opioids*, *Individuals with Multiple SUDs*, and *Individuals Who Inject Drugs*. These classes largely correspond to “individuals with limited polysubstance use”, “polysubstance users”, and “individuals primarily injecting heroin” described in a recent review (34). In the following, we describe the characteristics of each class and highlight their anticipated care needs.

### 4.1 Individuals primarily using opioids

Half of the sample was classified as *Individuals Primarily Using Opioids*, who did not exhibit clearly distinctive care preferences. While *Individuals with Multiple SUDs* predominantly engaged in treatment-oriented services—such as OAT and addiction counseling—and *Individuals Who Inject Drugs* primarily accessed low-threshold support, *Individuals Primarily Using Opioids* utilized both types of services. The intermediate position of this class between the other two groups, along with the absence of sharply distinctive features, may indicate unobserved heterogeneity and the existence of subgroups that are not fully captured by the selected indicator variables.

Compared to both other classes, a larger proportion of *Individuals Primarily Using Opioids* reported methadone or other opioids as their main substance of misuse. This may, in part, reflect the misclassification of prescribed OAT medications as substances of misuse. However, it could also indicate actual misuse, as previously reported (37, 38). A combination of both patterns is likely, although this cannot be conclusively disentangled.

The high proportion of “other opioids” as the main substance likely points to the misuse of prescription opioids, as the category includes analgesics such as tramadol, tilidine, and oxycodone (23). As shown in prior research, both OAT medications and prescription opioids are frequently used to manage withdrawal symptoms or to supplement perceived “insufficient” substitution doses (15, 39, 40). Consequently, a more rigorous assessment of the interplay between ongoing OAT, misuse of OAT medications, and misuse of prescription opioids is essential to better understand and address the care needs of *Individuals Primarily Using Opioids*.

### 4.2 Individuals with multiple SUDs

A quarter of the sample was classified as *Individuals with Multiple SUDs*, a group that is more likely to be embedded in comprehensive treatment, care, and support services (including addiction counseling, OAT, and psychosocial support) compared to the other classes. This comparatively strong connection to long-term addiction care may partly reflect the high likelihood of comorbid SUDs, indicative of a more complex clinical profile. Previous research has shown that polysubstance use is associated with more unmet subjective needs (41), which may in turn contribute to poorer treatment outcomes, including higher dropout and relapse rates (42, 43).

At the same time, strong social integration has been linked to fewer perceived needs among individuals engaging in polysubstance use (41), emphasizing the potential of interventions aimed at strengthening social support networks (44). This mechanism should be considered, as *Individuals with Multiple SUDs* in our sample showed relatively higher rates of independent living, partnership, and parenthood compared to the other classes, indicating a modest degree of social integration. These factors are thought to promote treatment adherence (45).

Notably, individuals in this class were less likely to have personal migration experience than observed in the other groups. While our data do not allow direct conclusions about access barriers, this observation is consistent with prior evidence suggesting that people with a first-generation migration background may encounter cultural or structural barriers (such as limited awareness of available services, language difficulties, and lack of health insurance) to comprehensive treatment services (13) to comprehensive treatment services might be more pronounced among people with a first-generation migration background, who appear to be underrepresented in addiction care. Indeed, previous evidence demonstrates that people with a migration background who use drugs encounter substantial barriers to treatment services (46). To help close the presumed gap in care provision, multilingual and culturally responsive offers appear paramount (47, 48).

### 4.3 Individuals who inject drugs

The last quarter of our sample was classified as *Individuals Who Inject Drugs*, a group frequently living in precarious housing conditions and primarily accessing low-threshold services. In these settings, comprehensive sociodemographic assessments are typically not prioritized, as the focus lies on offering immediate support, and providing harm reduction measures—such as needle and syringe exchange programs, naloxone distribution and training, and drug checking services—to reduce drug-related morbidity and mortality (49, 50). Therefore, the high level of missingness in socio-demographic parameters is highly plausible.

Injection drug use poses significant risks for blood-borne infections, vein damage, and overdose (51, 52). It is therefore crucial for OACFs to assess whether injection equipment is shared and to monitor clients for infections. This may account for the relatively low proportion of missing data on related variables.

Despite a substantial risk of drug-related harm, *Individuals Who Inject Drugs* seldom engage with professional care services, particularly OAT and addiction counseling. This may be partially explained by the double stigma resulting from both injection drug use (compared to other modes of consumption) and precarious housing, which together may promote a vicious cycle of limited access to care (53, 54). There remains a persistent need for targeted information campaigns, destigmatization efforts, and a more systematic rollout of outreach-based support services.

### 4.4 Practical implications

According to the World Health Organization, OAT is the most effective intervention for OUD, leading to substantial improvements in health and social outcomes. As such, it should be a central component of broader harm reduction and public health strategies (55).

Our analysis indicates that entering OAT is particularly challenging for help-seekers classified as *Individuals Who Inject Drugs*, who are likely to face multiple structural and social barriers to accessing professional care. Many individuals in this group live in unstable conditions and would benefit from improved coordination between health and social services, including housing and employment programs. Such integration could foster social reintegration and, in turn, improve access to treatment and care services (56).

For individuals already receiving OAT, tailoring treatment to their ongoing needs is essential. In response to the COVID-19 pandemic, several OAT regulations in Germany were revised to allow extended take-home prescriptions for less stable patients and the dispensing of medication without in-person contact (57, 58). Evidence from the UK suggests that some patients prefer more flexible, self-directed dosing of OAT medications (59). This may also apply to the group of *Individuals with Multiple SUDs* in our sample, who show relatively high levels of social integration and may need to balance occupational and family responsibilities with OAT.

For *Individuals Primarily Using Opioids*, more research is needed to understand their specific care needs and potential barriers to accessing OAT. Clarifying the interplay between prescribed OAT, non-prescribed use of substitution medication, and use of other prescription opioids will be essential for informing targeted strategies to improve engagement and optimize treatment outcomes.

### 4.5 Limitations and strengths

The results of our study should be interpreted considering several caveats. First, there may be underreporting of comorbid SUD diagnoses, particularly among help-seekers in low-threshold settings. In these contexts, healthcare providers may neither feel authorized nor sufficiently trained to conduct formal diagnostic assessments (23). Furthermore, the binary coding of diagnoses as either present or absent limits the ability to capture disorder severity, both for the primary OUD and for comorbid SUDs. This lack of diagnostic granularity may have affected the identification and interpretation of latent classes.

Another limitation relates to the assessment timeframe for injection drug use, which directly impacts prevalence estimates and model calculations. Shorter observation periods, such as 30 days, provide clinically meaningful data but may underestimate the extent of injection behavior. In contrast, lifetime measures offer greater statistical power but fail to reflect current risk exposure. The 12-month timeframe adopted in this study aims to balance these concerns by capturing sustained behaviors relevant to both health risks and treatment needs.

Finally, the available variables may not fully capture the multidimensional nature of social integration, marginalization, and health status. Indicators such as partnership, independent living, and employment provide only limited proxies for social functioning, while mental health comorbidities are insufficiently documented. As a result, the latent classes identified in this study may only partially reflect the complexity of help-seekers’ lived experiences.

Despite these limitations, the study has several notable strengths. It is the first to apply a person-centered approach to identify subgroups of help-seekers with OUD in OACFs in Germany using advanced analytical methods. Utilizing data from the BSHS enables a comprehensive examination of the local outpatient care landscape. This polycentric strategy reflects the heterogeneity of service delivery in urban settings and enables the identification of variations in access points and utilization patterns.

By focusing on help-seekers’ characteristics at the time of admission, the study provides relevant insights into the immediate social and health-related needs. Moreover, comparing these characteristics with broader profiles of individuals who use opioids offers a valuable opportunity to assess potential underrepresentation of specific subgroups in outpatient care. These findings may inform both the adaptation of existing services and the development of targeted outreach strategies for underserved populations.

## 5 Conclusion

Although the outpatient addiction care system in Berlin reaches both socially well-integrated and marginalized individuals with OUD, access to advanced treatment options is more common among the former. This underscores the persistent need to develop care structures that enable a seamless transition from low-threshold services to more comprehensive interventions—such as counseling and OAT—which is especially critical for high-risk and highly vulnerable subgroups.

Given the increasing prevalence of synthetic and prescription opioid consumption among individuals who use drugs, a re-evaluation of existing care models may be warranted to better address currently unmet needs.

Finally, in the absence of comprehensive data on subpopulations with OUD who are not yet reached, further qualitative research is needed to better understand their basic needs and perceived barriers to care and support.

## Data Availability

The datasets generated and analyzed during the current study are not publicly available due to data protection requirements. However, they can be made available upon request, subject to the signing of an individual data use agreement and the demonstration of reasonable scientific interest. Requests to access these datasets should be directed to schwarzkopf@ift.de.
