# Supplementary material for: Who are we reaching? Identifying subgroups among individuals seeking help for opioid use disorder
Source: Front Psychiatry. 2026 Mar 9;17:1753193. doi: 10.3389/fpsyt.2026.1753193 (PMC13006884; doi:10.3389/fpsyt.2026.1753193)
Supplement: Supplementary file 1 [file Supplementaryfile1.zip › Supplementary Figure 3-4.DOCX]

Supplementary Material

Sensitivity Analysis 1 presents the estimated class-specific response probabilities for the indicator variables based on the three-class model, with missing values treated as a separate category.

Notes. Sample Size was N = 2,960. IDU = Injection Drug Use; CoUD = Cocaine Use Disorder; PUD = Polysubstance Use Disorder; CUD = Cannabinoid Use Disorder; TUD = Tobacco Use Disorder; AUD = Alcohol Use Disorder. Higher scores represent a high probability of a particular indicator variable.

Supplementary Figure 3. Sensitivity Analysis 1

Sensitivity Analysis 2 presents the estimated class-specific response probabilities for the indicator variables based on the three-class model, including comorbid stimulant use disorder (F15.1, F15.2) as an additional indicator variable.

Notes. Sample Size was N = 2,806. IDU = Injection Drug Use; CoUD = Cocaine Use Disorder; PUD = Polysubstance Use Disorder; CUD = Cannabinoid Use Disorder; TUD = Tobacco Use Disorder; AUD = Alcohol Use Disorder. Higher scores represent a high probability of a particular indicator variable.

Supplementary Figure 4. Sensitivity Analysis 2
