# Supplementary material for: Who are we reaching? Identifying subgroups among individuals seeking help for opioid use disorder
Source: Front Psychiatry. 2026 Mar 9;17:1753193. doi: 10.3389/fpsyt.2026.1753193 (PMC13006884; doi:10.3389/fpsyt.2026.1753193)
Supplement: Supplementary file 1 [file Supplementaryfile1.zip › Supplementary Figure 5-6.DOCX]

Supplementary Material

Details on the 2-class model

Notes. Sample Size was N = 2,836. IDU = Injection Drug Use; CoUD = Cocaine Use Disorder; PUD = Polysubstance Use Disorder; CUD = Cannabinoid Use Disorder; TUD = Tobacco Use Disorder; AUD = Alcohol Use Disorder. Higher scores represent a high probability of a particular indicator variable.

Supplementary Figure 5. Estimated Class-Specific Response Probabilities for Indicator Variables (2-class model).

Details on the 4-class model

Notes. Sample Size was N = 2,694. IDU = Injection Drug Use; CoUD = Cocaine Use Disorder; PUD = Polysubstance Use Disorder; CUD = Cannabinoid Use Disorder; TUD = Tobacco Use Disorder; AUD = Alcohol Use Disorder. Higher scores represent a high probability of a particular indicator variable.

Supplementary Figure 6. Estimated Class-Specific Response Probabilities for Indicator Variables (2-class model).
