# Supplementary material for: Who are we reaching? Identifying subgroups among individuals seeking help for opioid use disorder
Source: Front Psychiatry. 2026 Mar 9;17:1753193. doi: 10.3389/fpsyt.2026.1753193 (PMC13006884; doi:10.3389/fpsyt.2026.1753193)
Supplement: Supplementary file 1 [file Supplementaryfile1.zip › Supplementary Figure 2.DOCX]

Supplementary Material

The Elbow plot is used to visualize the relationship between the number of classes and model fit indices, such as BIC and SABIC. The plot typically shows a decrease in the fit indices as the number of classes increases, followed by a point where the rate of improvement slows down. This "elbow" indicates the optimal number of classes, as adding more classes beyond this point yields only marginal improvements in model fit. In this analysis, the BIC and SABIC are plotted to assess the trade-off between model complexity and fit. When the slope flattens, it is recommended to closely inspect the models with one more and one fewer class to ensure the most appropriate class solution is selected.

**Supplementary Figure 2.** Elbow Plot of Model Fit Indices
