# Supplementary material for: Who are we reaching? Identifying subgroups among individuals seeking help for opioid use disorder
Source: Front Psychiatry. 2026 Mar 9;17:1753193. doi: 10.3389/fpsyt.2026.1753193 (PMC13006884; doi:10.3389/fpsyt.2026.1753193)
Supplement: Supplementary file 1 [file Supplementaryfile1.zip › Supplementary Figure 1.DOCX]

Supplementary Material

Supplementary Figure 1. Flowchart of Participants
